# The effects of tumor resection and adjuvant therapy on the peripheral blood immune cell profile in patients with colon carcinoma

**DOI:** 10.1007/s00262-020-02590-z

**Published:** 2020-05-12

**Authors:** Daniëlle Krijgsman, Natasja L. De Vries, Morten N. Andersen, Anni Skovbo, Rob A. E. M. Tollenaar, Esther Bastiaannet, Peter J. K. Kuppen, Marianne Hokland

**Affiliations:** 1 Department of Surgery, Leiden University Medical Center, Albinusdreef 2, 2300 RC Leiden, The Netherlands; 2 Department of Biomedicine, Aarhus University, Aarhus, Denmark; 3 FACS Core Facility, Aarhus University, Aarhus, Denmark; 4 Department of Clinical Biochemistry, Aarhus University Hospital, Aarhus, Denmark; 5 Present Address: Department of Immunohematology and Blood Transfusion, Leiden University Medical Center, Leiden, The Netherlands

**Keywords:** Colon carcinoma, Peripheral blood immune cell profile, Cancer immunology, Natural cytotoxicity receptors, Adjuvant therapy, Tumor resection

## Abstract

**Objective:**

The subset distribution and immunophenotype of circulating immune cells (“peripheral blood immune cell profile”) may reflect tumor development and response to cancer treatment. In order to use the peripheral blood immune cell profile as biomarker to monitor patients over time, it is crucial to know how immune cell subsets respond to therapeutic interventions. In this study, we investigated the effects of tumor resection and adjuvant therapy on the peripheral blood immune cell profile in patients with colon carcinoma (CC).

**Methods:**

The subset distribution and immunophenotype of T cells (CD3^+^CD56^−^), CD56^dim^ NK cells (CD3^−^CD56^dim^), CD56^bright^ NK cells (CD3^−^CD56^bright^) and NKT-like cells (CD3^+^CD56^+^) were studied in preoperative and postoperative peripheral blood mononuclear cell (PBMC) samples of 24 patients with CC by multiparameter flow cytometry. Changes in immunophenotype of circulating immune cells after tumor resection were studied in patients treated with and without (capecitabine-based) adjuvant therapy.

**Results:**

The NKT-like cell (% of total PBMCs) and CD8^+^ T cell (% of total T cells) populations expanded in the peripheral blood of non-adjuvant-treated CC patients after surgery. NK- and NKT-like cells showed upregulation of activating receptors and downregulation of inhibitory receptors in non-adjuvant-treated CC patients after surgery. These changes were not observed in the peripheral blood of adjuvant-treated CC patients.

**Conclusions:**

Our results suggest tumor-induced suppression of NK- and NKT-like cells in CC patients, an effect that could not be detected after tumor resection. In contrast, adjuvant therapy maintained tumor-induced immunosuppression of NK- and NKT-like cells in CC patients.

## Introduction

Colon carcinoma (CC) is a tumor type with a high incidence and, combined with rectal cancer, globally accounting for more than 1 million new cases and around 600.000 deaths annually [1–3]. Despite improved surgical procedures and adjuvant treatment strategies, around 30% of the patients, without metastatic disease at time of diagnosis, develop recurrence or dissemination of the disease following successful resection of the primary tumor [4]. Currently, different biomarkers are evaluated for their prognostic value that may be used to identify cancer patients at risk of disease progression. Hence, biomarker application in cancer patients is crucial since it may provide information for therapeutic decision making, including decisions on the frequency of clinical follow-up, thereby possibly prolonging survival and reducing the risk of recurrence in cancer patients. Over the past decades, it has become clear that immunosurveillance plays a pivotal role in colon cancer by protecting the host against tumor development and progression. For instance, the immunoscore, representing CD3^+^/CD8^+^ T lymphocyte density in tumor tissue, is a strong prognostic factor in colorectal cancer (CRC) [5–9]. Low density of cytotoxic (CD8^+^) tumor-infiltrating T cells was reported to correlate with poor clinical outcome in CRC [5, 6, 10, 11]. However, it must be realized that the immunoscore can only be determined once at time of surgery because it is performed on resection material. Therefore, it does not provide information regarding treatment response of patients over time. In general, immune cells are present in relatively low numbers in the tissue of primary tumors, whereas they are abundantly present in peripheral blood. Interestingly, the subset distribution and immunophenotype of circulating immune cells (“peripheral blood immune cell profile”) are associated with clinical outcome of CRC patients [12]. In contrast to the immunoscore, the peripheral blood immune cell profile can be monitored in patients over time since it only requires blood sampling.

The peripheral blood comprises different immune cell subsets, among which are natural killer (NK) cells that can be subdivided based on their CD56 expression. CD56^dim^ NK cells have an important cytotoxic function, while CD56^bright^ NK cells are generally associated with production of pro-inflammatory cytokines and immunoregulatory properties [13, 14]. Furthermore, peripheral blood contains a unique natural killer T (NKT) cell population with characteristics of both NK cells and NKT cells. Co-expression of CD3 and CD56 can be used to identify this immune subset in the circulation using flow cytometry, which is often referred to as “NKT-like” [15]. Like other immune cell subsets, NK- and NKT-like cell activity is dependent on a delicate balance between activating and inhibitory signals from cell surface receptors [16]. The activating signals are mediated by a wide array of receptors including natural killer group 2-C (NKG2C), natural killer group 2-D (NKG2D), DNAX accessory molecule-1 (DNAM-1), CD161, and natural cytotoxicity receptors NKp30, NKp44, and NKp46 that recognize a variety of stress-induced molecules that may be present on tumor cells. Additionally, the CD16 (FcγRIII) receptor mediates antibody-dependent cell-mediated cytotoxicity and CD8 enhances the cytolytic activity of NK cells [17, 18]. NK cell inhibitory receptors include natural killer group 2-A (NKG2A) and killer cell immunoglobulin (Ig)-like receptors CD158a and CD158b that recognize human leukocyte antigen (HLA) class I molecules. Recently, we [12] and others [19–21] have shown that the peripheral blood immune cell profile is altered in CRC patients compared to healthy donors, characterized by downregulation of activating receptors on circulating CD56^dim^ NK cells and NKT-like cells.

Several studies reported a critical role for the tumor microenvironment (TME) in shaping NK- and NKT-like receptor-mediated anti-tumor immunity [15, 22, 23]. For instance, immune-modulating effects of NK cells have been attributed to hypoxic conditions [24] and immunosuppressive cytokines and signaling molecules [23, 25, 26] present in the TME. As a result of these immune modulations, NK- and NKT-like cells may downregulate activating receptors and, as a result, may become functionally impaired, thereby promoting tumor escape. Up till now, it is unclear whether TME-induced immune-modulations of circulating NK- and NKT-like cells are reversible. Furthermore, chemotherapy, often used for adjuvant treatment of CRC patients, has been reported to have an immunosuppressive effect on the immune system, including overall impairment of NK cell responses [27]. In order to use the peripheral blood immune cell profile as biomarker to monitor cancer patients over time, it is crucial to know how immune cell subsets respond to therapeutic interventions. In a recent paper [12], we studied the preoperative peripheral blood immune cell profile in patients with CRC. In this study, we investigated the peripheral blood immune cell profile in patients with available postoperative blood samples in order to study the effects of tumor resection and adjuvant therapy. It was not possible to study the effects of tumor resection and adjuvant therapy in patients with rectum tumors due to limited sample availability. Therefore, we focused on patients with colon tumors. Additionally, we selected patients with Tumor–node–metastasis (TNM) stage II and III who are at risk for development of metastases after surgery and are, therefore, the patients that would qualify for biomarker monitoring over time. In summary, our collection of peripheral blood mononuclear cells (PBMCs) before and after tumor resection enabled, for the first time, to study the effects of tumor resection as well as adjuvant therapy on the subset distribution and immunophenotype of circulating T-, NK- and NKT-like cells in CC patients.

## Materials and methods

### Study population

The study population comprised patients diagnosed with CC who underwent surgical resection of their primary tumor at the Leiden University Medical Center (LUMC, the Netherlands) between 2001 and 2007. Patients were included in this study based on availability of PBMC samples. PBMC samples were collected within a month prior to surgery and ≥ 2 months after surgery (mean 8.2 months after surgery, range 2.2–17.7). When the patient started adjuvant therapy directly after surgery, postoperative PBMC samples were obtained after therapy completion. These samples were obtained ≥ 5 months after the final therapy date (mean 8.4 months after surgery, range 6.7–13.7). Most adjuvant therapy cycles consisted of 3 weeks of therapy; therefore, the included postoperative PBMC samples were obtained ≥ 4 months after therapy completion. In the present study, patients with histologically proven primary CC, TNM stage II and III, surgical R0 resection, and a minimal amount of 5 million cryopreserved preoperative as well as postoperative PBMCs were included (*N* = 26). Clinicopathological data of all patients were available. All blood samples were obtained after approval by the Medical Ethical Committee of the LUMC (protocol number P000.193). All procedures performed in this study were in accordance with the ethical standards of the Dutch law (“WMO”, medical research involving human subjects act), and with the 1964 Helsinki declaration and its later amendments or comparable ethical standards. All CC patients included in this study agreed to our use of their PBMCs and data for research purposes prior to blood sampling via written informed consent, and they moreover agreed to anonymous publication of the resulting data.

### Isolation of peripheral blood mononuclear cells

PBMCs from CC patients were isolated and cryopreserved as previously described [12]. PBMCs were also isolated from the buffy coat of a random blood donor obtained from the Blood Bank at Aarhus University Hospital (Dept. Clinical Immunology, Aarhus University Hospital, Skejby, Denmark), which was used as an internal control in the flow cytometry experiments [12].

### Flow cytometry antibody staining and data analysis

All preoperative PBMC samples from CC patients were previously immunophenotyped and reported on as part of a CRC study [12]. All postoperative PBMC samples selected for the current study were immunophenotyped following the same protocol. Briefly, PBMC samples were thawed following a standard protocol and the cell concentration was adjusted to 10 × 10^6^ cells/mL. PBMCs were then blocked for 15–30 min at room temperature with 50 μg/mL human IgG (CSL Behring, Bern, Switzerland) and subsequently stained with two distinct antibody panels containing selected NK- and NKT cell markers as previously described [12]. After the staining procedure, samples were fixed in PBS supplemented with 0.9% formaldehyde (Sigma-Aldrich, St. Louis, MO) and analyzed the same day by flow cytometry on a 4-laser equipped LSRFortessa (BD Biosciences, San Diego, CA) using Diva 7.0 software (BD Biosciences). Results were analyzed with FlowJo software v10.1 (Tree Star Inc., Ashland, OR). The buffy coat was used as an internal control in order to check for any inter-experimental variation. Expression of phenotypic markers on peripheral blood T-, NK- and NKT-like cells was evaluated by the median fluorescence intensity (MFI) and/or the percentage of positive cells. A compensation matrix was calculated to compensate for spillover of signal into other channels in the multicolor flow cytometry experiments. This matrix was set up using single stains on CompBeads (BD Biosciences,) OneComp eBeads (eBioscience, San Diego, CA), CompBeads Plus (BD Biosciences) and ArC reactive beads (Life Technologies). A standardized gating strategy based on a healthy donor buffy coat was used in order to study the distribution of markers on specific lymphocyte subsets as described previously [12].

### Statistical analyses

Statistical analyses were conducted using SPSS statistical software (IBM SPSS Statistics 23, Chicago, USA). The age of patients treated with adjuvant therapy was compared with the age of patients treated without adjuvant therapy using the Mann–Whitney U test. Preoperative and postoperative PBMC samples from patients were compared using the paired samples T test and Wilcoxon signed-rank test, for normally distributed and not normally distributed variables, respectively. We corrected for multiple testing using the Benjamini–Hochberg method, by which adjusted *P*-values were calculated (indicated by P*) [28]. P*-values ≤ 0.05 were considered statistically significant.

## Results

### Study population

The subset distribution and immunophenotype of circulating T-, NK- and NKT-like cells were studied pre- and postoperatively in patients diagnosed with CC TNM stage II and III (*N* = 26). One postoperative sample was excluded due to low viability of the PBMCs (< 50% viable cells). Additionally, one patient was diagnosed with Lynch syndrome. Since tumors from patients with Lynch syndrome are immunologically different compared to tumors from patients with sporadic cancer [29], this patient was excluded from analyses, resulting in a total study population of 24 patients. One patient had undergone local radiotherapy prior to the collection of the preoperative PBMC sample. In total, 15 patients underwent surgical resection of their tumor only (non-adjuvant-treated group) and 9 patients received additional therapy after surgery (adjuvant-treated group). Two of these 9 patients were treated with capecitabine monotherapy, six patients were treated with a combination of capecitabine and oxaliplatin, and one patient was treated with a combination of capecitabine, oxaliplatin and bevacizumab. Table 1 summarizes the clinicopathological characteristics of the 24 patients included in the analyses. Patients undergoing adjuvant therapy were younger (median 56 years) compared to patients that did not receive adjuvant therapy (median 73 years, *P* = 0.002). The low number of patients did not allow for analyzing the peripheral blood immune cell profile according to age.

**Table 1:** Patient demographics and tumor characteristics. Clinicopathological data of the 24 CC patients in this study

	CC patients	CC patients	CC patients
	Total population	No adjuvant therapy	Adjuvant therapy^a^
	(*N* = 24)	(*N* = 15)	(*N* = 9)
*Age at time of surgery*			
Median (years)	65	73	56
Range (years)	25–85	56–85	25–71
*Sex*			
Female	11 (45.8%)	7 (46.7%)	4 (44.4%)
Male	13 (54.2%)	8 (53.3%)	5 (55.6%)
*TNM classification*			
Stage II	12 (50.0%)	10 (66.7%)	2 (22.2%)
Stage III	12 (50.0%)	5 (33.3%)	7 (77.8%)
*Tumor differentiation*			
Well/moderate	21 (87.5%)	12 (80.0%)	9 (100%)
Poor	3 (12.5%)	3 (20.0%)	0 (0%)
*Neoadjuvant radiotherapy*			
Yes	1 (4.2%)	1 (6.7%)	0 (0%)
No	23 (95.8%)	14 (93.3%)	9 (100%)

^a^Capecitabine monotherapy, capecitabine with oxaliplatin, or a combination of capecitabine, oxaliplatin and bevacizumab

*CC* Colon carcinoma, *TNM* Tumor–node–metastasis

### Expansion of NKT-like and CD8^+^ T cell populations in peripheral blood after surgery in non-adjuvant-treated CC patients

**Fig.1:**
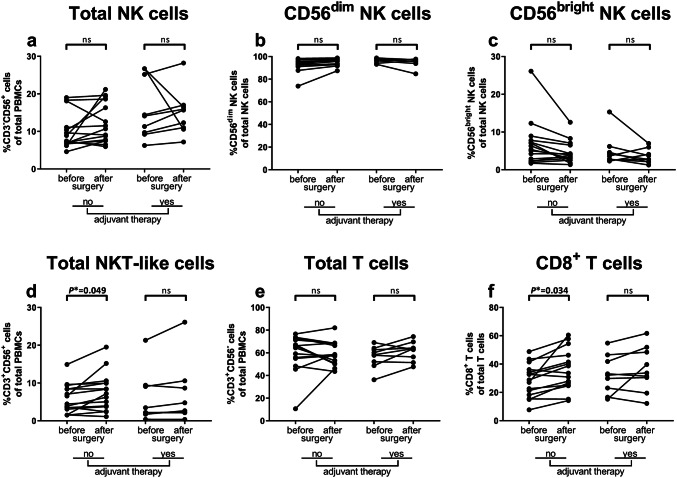
Effects of tumor resection and adjuvant therapy on the peripheral blood immune cell subset distribution in CC patients. Multiparameter flow cytometry was used to study the peripheral blood immune profile in PBMC samples from CC patients. The distribution of circulating immune cell subsets was compared before and after surgery in CC patients that did (*N* = 9) or did not (*N* = 15) receive adjuvant therapy. **a** Total percentage of NK cells (% CD3^−^CD56^+^ cells of total PBMCs). **b** Percentage of CD56^dim^ NK cells (% of total NK cells). **c** Percentage of CD56^bright^ NK cells (% of total NK cells). **d** Total percentage of NKT-like cells (% CD3^+^CD56^+^ cells of total PBMCs). **e** Total percentage of T cells (% CD3^+^CD56^−^ cells of total PBMCs). **f** Percentage of CD8^+^ T cells (% of total T cells). Pre- and postoperative samples from each patient are connected with a line. P*-values ≤ 0.05 were considered statistically significant. *CC* Colon carcinoma, *PBMCs* peripheral blood mononuclear cells, *NK* natural killer, *NKT* natural killer T, *ns* not significant

First, the effects of tumor resection and adjuvant therapy on the distribution of peripheral blood immune subsets were studied in non-adjuvant-treated (*N* = 15) and adjuvant-treated (*N* = 9) CC patients (Table 2). The percentages of total NK cells (% CD3^−^CD56^+^ cells of total PBMCs), CD56^dim^ NK cells (% of total NK cells), and CD56^bright^ NK cells (% of total NK cells) did neither change in non-adjuvant-treated patients nor in adjuvant-treated patients after resection of the primary tumor (Fig. 1a–c, respectively). The percentage of NKT-like cells (% CD3^+^CD56^+^ cells of total PBMCs) increased (P* = 0.049) in non-adjuvant-treated patients after surgery (Fig. 1d). Furthermore, although the percentage of total T cells (% CD3^+^CD56^−^ cells of total PBMCs) was not altered in non-adjuvant-treated patients after resection of the primary tumor (Fig. 1e), the percentage of CD8^+^ T cells (% of total T cells) was significantly increased (P* = 0.034, Fig. 1f). No change was observed in the percentage of NKT-like cells or CD8^+^ T cells in adjuvant-treated patients after surgery.

**Table 2:** Effects of tumor resection and adjuvant therapy on the peripheral blood immune cell profile in CC patients. The peripheral blood immune profile was studied in preoperative and postoperative PBMC samples from CC patients treated without (*N* = 15) and with (*N* = 9) adjuvant therapy using multiparameter flow cytometry. Statistically significant P*-values are indicated in bold

	No adjuvant therapy		No adjuvant therapy			Adjuvant therapy		Adjuvant therapy		
	Preoperative CC patients		Postoperative CC patients			Preoperative CC patients		Postoperative CC patients		
	(*N* = 15)		(*N* = 15)			(*N* = 9)		(*N* = 9)		
	Mean	SD	Mean	SD	P*-value	Mean	SD	Mean	SD	P*-value
*Subset distribution*										
T cells (%)	60.3	16.7	89.8	10.4	0.586^ W^	56.5	9.8	61.5	8.4	0.369^ W^
CD8^+^ T cells (%)	28.4	11.7	35.8	14.4	**0.034**^** T**^	32.6	13.4	36.6	15.7	0.455^ T^
NK cells (%)	9.9	4.8	12.2	5.4	0.148^ W^	16.0	8.1	14.9	6.0	0.657^ W^
CD56^dim^ NK cells (%)	92.2	6.1	95.4	3.0	0.083^ W^	96.5	1.9	95.0	4.1	0.535^ W^
CD56^bright^ NK cells (%)	6.8	6.1	4.6	3.0	0.090^ W^	3.5	1.9	4.9	4.1	0.514^ W^
NKT-like cells (%)	5.8	3.8	7.6	5.0	**0.049**^ W^	5.8	6.7	6.6	8.0	0.494^ W^
*CD56*^*dim*^* NK cells*										
CD16^+^ (%)	80.0	12.4	82.7	6.0	0.988^ W^	81.0	11.8	84.8	6.2	0.651^ W^
CD158a^+^ (%)	28.5	14.9	27.0	14.2	0.085^ T^	37.1	20.9	34.6	20.2	0.436^ T^
CD158b^+^ (%)	37.8	13.6	38.2	13.5	0.993^ W^	34.4	14.3	32.9	15.3	1.003^ W^
NKG2A^+^ (%)	48.9	18.6	43.8	16.6	0.133^ T^	38.6	13.8	40.3	13.3	0.551^ T^
NKG2A^+^ (MFI)	9042	3049	7929	3827	0.086^ W^	5726	1932	6935	2156	0.253^ W^
NKG2C^+^ (%)	21.8	15.2	22.7	16.5	0.972^ W^	17.5	20.0	19.3	20.0	0.615^ W^
NKG2C^+^ (MFI)	4070	3242	4614	3829	0.085^ W^	4238	5774	4338	5496	0.503^ W^
CD161 (MFI)	2923	1263	2820	1124	0.557^ T^	2848	1463	3025	1369	0.638^ T^
CD8^+^ (%)	23.8	8.8	24.0	9.2	0.862^ W^	30.7	20.7	37.6	17.9	0.240^ W^
CD8^+^ (MFI)	2899	1333	3853	1120	0.060^ W^	3850	1343	4370	1582	0.561^ W^
DNAM-1 (MFI)	517	155	540	148	0.562^ T^	516	140	572	131	0.305^ T^
NKG2D^+^ (%)	89.6	6.5	91.2	3.8	0.439^ W^	90.5	5.0	91.0	4.0	0.986^ W^
NKG2D^+^ (MFI)	3717	1344	4475	1037	**0.045**^** T**^	3452	1247	4015	778	0.483^ T^
NKp30 (MFI)	1482	963	1423	933	0.807^ T^	1442	570	1755	802	0.327^ T^
NKp44^+^ (%)	0.5	0.3	0.7	0.4	0.132^ W^	0.7	0.5	0.7	0.3	0.488^ W^
NKp44 (MFI)	118	28	145	10	**0.036**^** T**^	133	25	145	15	0.420^ T^
NKp46^+^ (%)	36.2	19.9	34.6	17.0	0.803^ T^	36.2	17.7	45.5	18.6	0.482^ T^
NKp46 (MFI)	529	313	500	295	0.846^ W^	510	239	671	354	0.496^ W^
*CD56*^*bright*^* NK cells*										
CD16^+^ (%)	2.8	1.8	1.9	1.4	**0.040**^** W**^	1.6	1.1	2.9	2.8	0.344^ W^
CD158a^+^ (%)	5.0	3.1	5.8	3.1	0.519^ W^	9.7	6.8	7.8	5.9	0.753^ W^
CD158b^+^ (%)	9.0	8.7	10.0	7.2	0.596^ W^	8.8	5.1	7.9	7.3	0.969^ W^
NKG2A^+^ (%)	5.5	3.1	4.3	3.2	0.224^ W^	3.1	2.0	4.6	4.1	0.494^ W^
NKG2A^+^ (MFI)	21,772	6987	18,008	5705	**0.045**^** W**^	17,143	4384	19,518	5110	0.473^ W^
NKG2C^+^ (%)	1.5	1.4	2.1	2.2	0.829^ W^	1.1	0.7	1.7	1.1	0.476^ W^
NKG2C^+^ (MFI)	2956	2358	3007	2901	0.928^ W^	2414	1675	2515	1882	0.740^ W^
CD161 (MFI)	1709	752	1852	550	0.295^ T^	2105	572	2177	553	0.686^ T^
CD8^+^ (%)	1.9	1.3	1.5	1.1	0.505^ W^	1.2	0.7	2.4	2.1	0.348^ W^
CD8^+^ (MFI)	3085	1629	3677	1365	0.051^ W^	3379	715	3720	1551	0.644^ W^
DNAM-1 (MFI)	790	183	806	154	0.775^ T^	781	139	877	116	0.240^ T^
NKG2D^+^ (%)	6.2	5.4	4.0	2.4	0.103^ W^	3.4	1.9	4.8	3.9	0.459^ W^
NKG2D^+^ (MFI)	7064	2766	8248	1954	0.051^ T^	6379	1973	7096	1501	0.501^ T^
NKp30 (MFI)	2134	572	2271	509	0.434^ T^	2231	507	2524	719	0.309^ T^
NKp44^+^ (%)	1.3	0.8	1.4	1.2	1.000^ W^	1.2	1.1	1.7	1.1	0.472^ W^
NKp44 (MFI)	222	61	270	47	0.081^ W^	295	141	306	63	0.451^ W^
NKp46^+^ (%)	79.7	16.5	78.2	15.5	0.725^ W^	75.6	20.9	89.0	5.7	0.225^ W^
NKp46 (MFI)	2237	1030	2184	482	0.813^ W^	1893	762	2753	818	0.330^ W^
*NKT-like cells*										
CD16^+^ (%)	21.1	13.4	20.2	15.1	0.263^ W^	16.7	9.6	26.5	14.6	0.210^ W^
CD158a^+^ (%)	8.6	11.1	7.4	10.1	**0.048**^** W**^	10.4	12.5	12.6	12.9	0.601^ W^
CD158b^+^ (%)	23.8	26.3	21.1	24.2	0.161^ W^	8.7	4.7	11.0	5.2	0.417^ W^
NKG2A^+^ (%)	21.1	18.1	15.3	14.2	**0.030**^** W**^	33.2	17.7	30.6	14.5	0.822^ W^
NKG2A^+^ (MFI)	3334	3813	6250	5937	0.081^ W^	7398	4106	10,845	4848	0.220^ W^
NKG2C^+^ (%)	15.1	14.0	13.3	12.2	0.895^ W^	14.4	13.0	14.1	13.1	0.547^ W^
NKG2C^+^ (MFI)	2657	2160	3029	3185	0.971^ W^	1694	2488	1685	2403	0.631^ W^
CD161 (MFI)	2126	1796	2055	1689	0.975^ W^	7309	9278	4287	3896	0.459^ W^
CD8^+^ (%)	76.4	16.4	77.8	15.5	0.255^ W^	75.5	16.4	76.7	17.3	0.618^ W^
CD8^+^ (MFI)	11,727	5260	15,077	6501	**0.030**^** T**^	15,142	6583	18,619	9320	0.342^ T^
DNAM-1 (MFI)	726	329	730	381	0.974^ T^	712	191	876	211	0.210^ T^
NKG2D^+^ (%)	92.0	9.0	92.5	8.5	0.098^ W^	91.8	7.4	92.5	8.3	0.663^ W^
NKG2D^+^ (MFI)	6176	2776	7155	2225	**0.048**^** T**^	4836	2465	6413	2180	0.192^ T^
NKp30 (MFI)	235	159	157	26	0.468^ W^	183	65	182	60	0.953^ W^
NKp44^+^ (%)	1.1	0.6	2.1	0.7	**0.040**^** W**^	2.2	1.5	2.5	1.1	0.588^ W^
NKp44 (MFI)	122	34	156	12	**0.038**^** W**^	147	45.8	152	13.3	0.490^ W^
NKp46^+^ (%)	3.8	1.0	3.1	1.0	0.081^ W^	3.0	1.3	4.3	2.1	0.349^ W^
NKp46 (MFI)	94	41	84	31	0.570^ T^	56	25	69.0	31	0.506^ T^

^T^Paired samples *T* test

^W^Wilcoxon signed-rank test

*CC* Colon carcinoma, *MFI* median fluorescence intensity, *NK* natural killer, *NKT* natural killer T, *SD* standard deviation

### Increased expression of activating receptors and downregulation of inhibitory receptors on NK cells in peripheral blood after surgery in non-adjuvant-treated CC patients

**Fig. 2:**
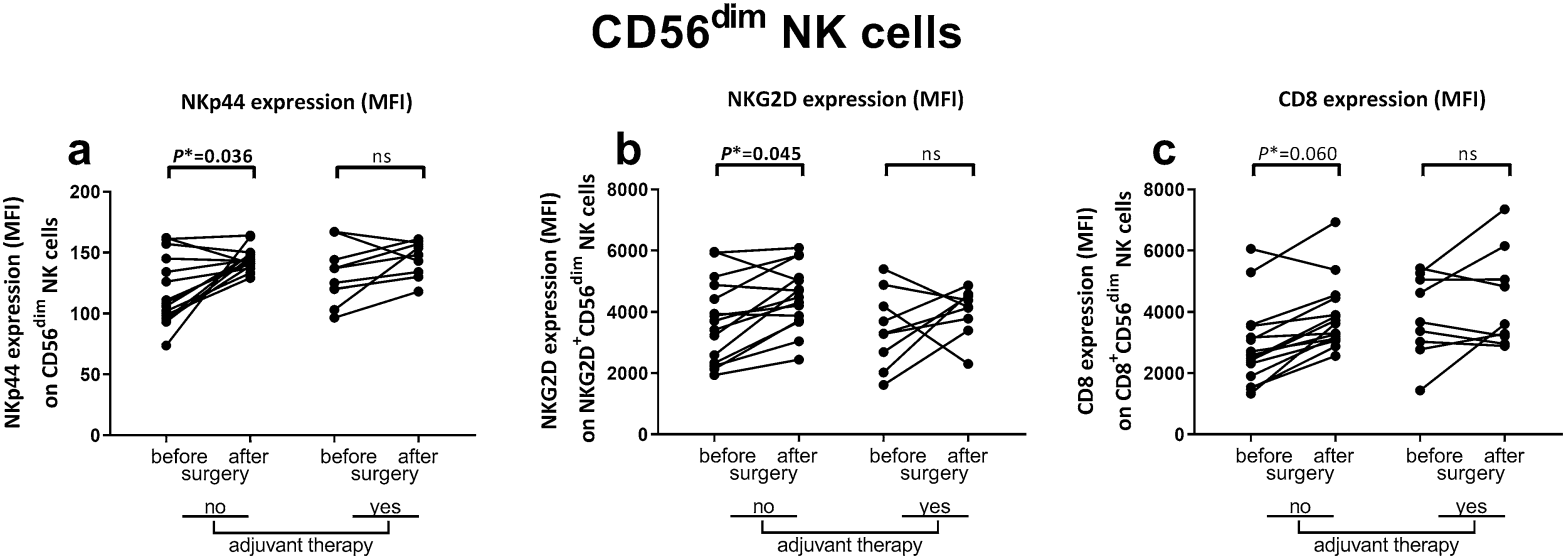
Effects of tumor resection and adjuvant therapy on the immunophenotype of CD56^dim^ NK cells in CC patients. Multiparameter flow cytometry was used to study the peripheral blood immune profile in PBMC samples from CC patients. The immunophenotype of CD56^dim^ NK cells was compared before and after surgery in CC patients that did (*N* = 9) or did not (*N* = 15) receive adjuvant therapy. **a** Expression level of NKp44 on CD56^dim^ NK cells. **b** Expression level of NKG2D on NKG2D^+^CD56^dim^ NK cells. **c** Expression level of CD8 on CD8^+^CD56^dim^ NK cells. Pre- and postoperative samples from each patient are connected with a line. Statistically significant P*-values are indicated in bold. *CC* Colon carcinoma, *MFI* median fluorescence intensity, *NK* natural killer, *ns* not significant

**Fig. 3:**
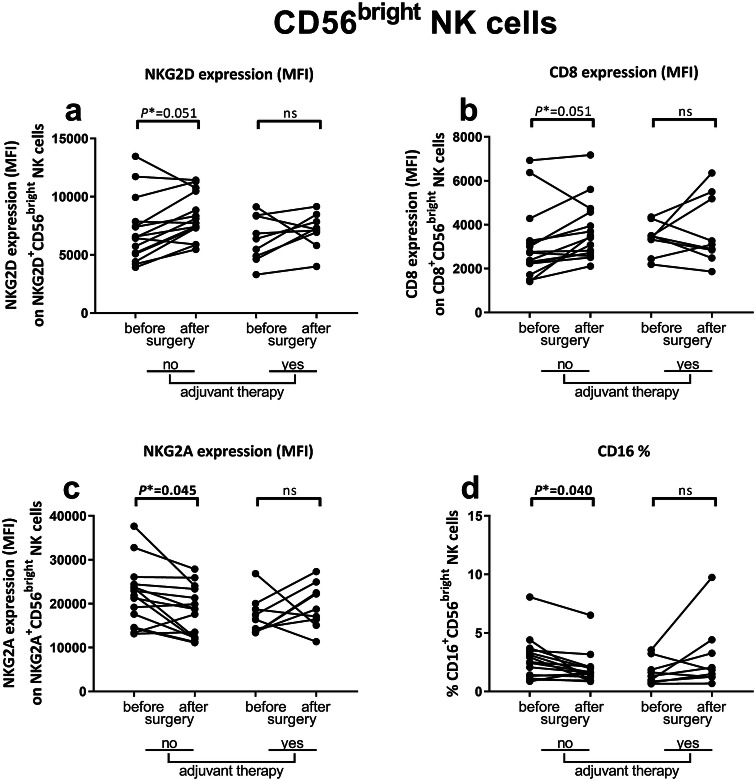
Effects of tumor resection and adjuvant therapy on the immunophenotype of CD56^bright^ NK cells in CC patients. Multiparameter flow cytometry was used to study the peripheral blood immune profile in PBMC samples from CC patients. The immunophenotype of CD56^bright^ NK cells was compared before and after surgery in CC patients that did (*N* = 9) or did not (*N* = 15) receive adjuvant therapy. **a** Expression level of NKG2D on NKG2D^+^CD56^bright^ NK cells. **b** Expression level of CD8 on CD8^+^CD56^bright^ cells. **c** Expression level of NKG2A on NKG2A^+^CD56^bright^ NK cells. **d** Percentage of CD16^+^CD56^bright^ NK cells. Pre- and postoperative samples from each patient are connected with a line. Statistically significant P*-values are indicated in bold. *CC* Colon carcinoma, *MFI* median fluorescence intensity, *NK* natural killer, *ns* not significant

After analyzing the distribution of immune cell subsets, we compared the immunophenotype of NK- and NKT-like cells before and after tumor resection in non-adjuvant-treated and adjuvant-treated CC patients (Table 2). Within the CD56^dim^ NK cell population, the expression levels of activating receptors NKp44 (P* = 0.036) and NKG2D (P* = 0.045) increased after surgery in non-adjuvant-treated patients (Fig. 2a, b, respectively). Additionally, a trend was observed towards a higher expression level of the co-stimulatory molecule CD8 in non-adjuvant-treated patients after surgery (P* = 0.060) (Fig. 2c). The expression levels of NKp44, NKG2D and CD8 did not change on CD56^dim^ NK cells after tumor resection in adjuvant-treated patients (Fig. 2a–c, respectively). In line with these results, a trend towards increased expression levels of NKG2D (P* = 0.051) and CD8 (P* = 0.051) was observed on CD56^bright^ NK cells in non-adjuvant-treated patients (Fig. 3a, b, respectively). No change in expression levels of NKG2D and CD8 was observed on CD56^bright^ NK cells after tumor resection in adjuvant-treated patients. The expression level of the inhibitory receptor NKG2A (P* = 0.045) on CD56^bright^ cells decreased in non-adjuvant-treated patients after surgery (Fig. 3c). In contrast, the percentage of stimulatory receptor CD16^+^ CD56^bright^ NK cells was also decreased (P* = 0.040) in non-adjuvant-treated patients after tumor resection (Fig. 3d). The expression level of NKG2A and the percentage of CD16^+^ cells did not change after surgery in adjuvant-treated patients. In summary, circulating NK cells acquired expression of cell surface markers associated with functional activity after surgery in non-adjuvant-treated CC patients as compared to before surgery.

### Increased expression of activating receptors and downregulation of inhibitory receptors on NKT-like cells in peripheral blood after surgery in non-adjuvant-treated CC patients

**Fig. 4:**
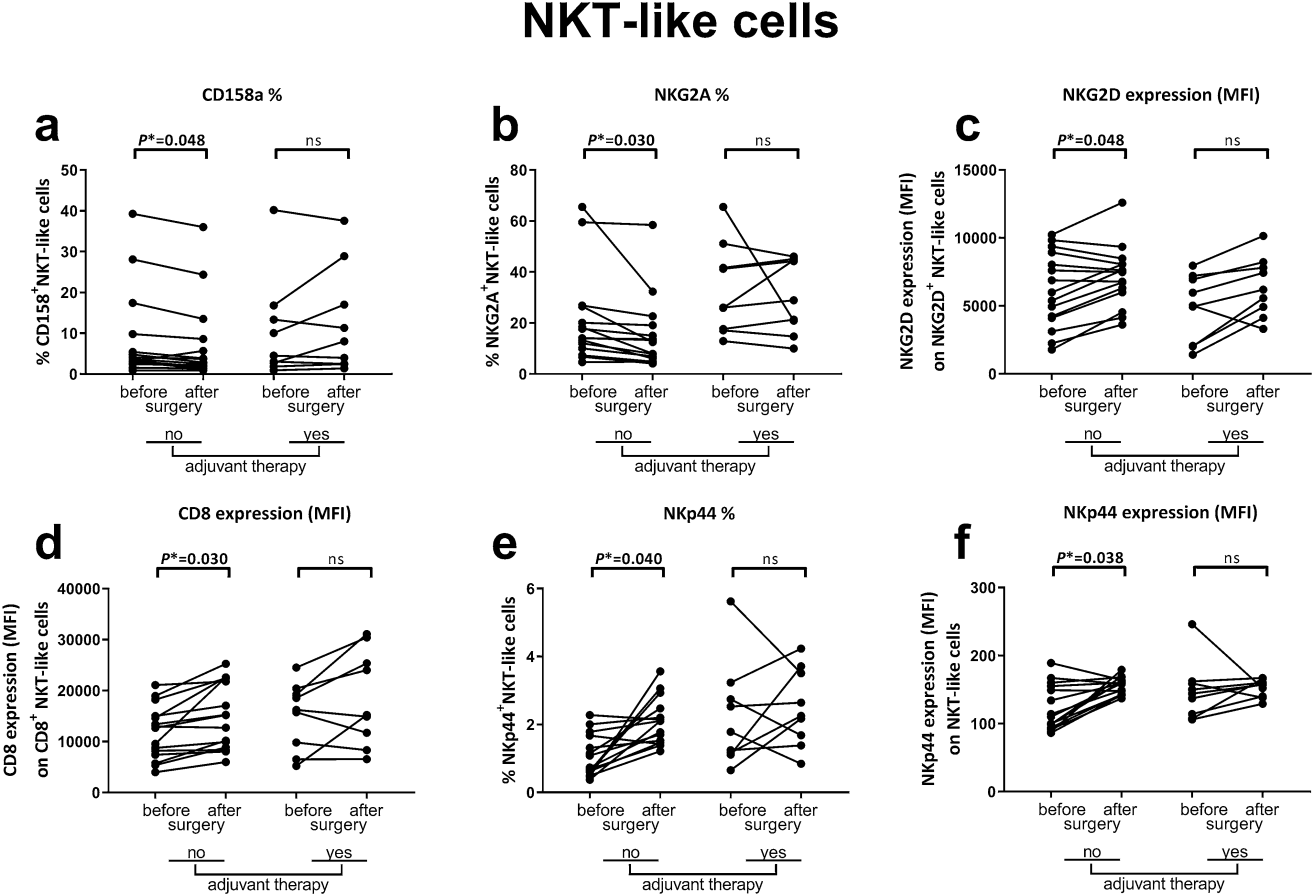
Effects of tumor resection and adjuvant therapy on the immunophenotype of NKT-like cells in CC patients. Multiparameter flow cytometry was used to study the peripheral blood immune profile in PBMC samples from CC patients. The immunophenotype of NKT-like cells was compared before and after surgery in CC patients that did (*N* = 9) or did not (*N* = 15) receive adjuvant therapy. **a** Percentage of CD158a^+^NKT-like cells. **b** Percentage of NKG2A^+^NKT-like cells. **c** Expression level of NKG2D on NKG2D^+^NKT-like cells. **d** Expression level of CD8 on CD8^+^NKT-like cells. **e** Percentage of NKp44^+^NKT-like cells. **f** Expression level of NKp44 on NKT-like cells. Pre- and postoperative samples from each patient are connected with a line. Statistically significant P*-values are indicated in bold. *CC* Colon carcinoma, *CI* confidence interval, *MFI* median fluorescence intensity, *NKT* natural killer T, *ns* not significant

Within the NKT-like cell subset, the percentages of NKT-like cells expressing the inhibitory receptors CD158a or NKG2A decreased in non-adjuvant-treated patients after surgery (P* = 0.048 and P* = 0.030, respectively), whereas no change was observed in adjuvant-treated patients (Fig. 4a, b). In contrast, the expression levels of activating receptors NKG2D (P* = 0.048) and co-stimulatory receptor CD8 (P* = 0.030) on NKT-like cells increased in non-adjuvant-treated patients after surgery (Fig. 4c, d). No change in NKT-like cell expression levels of NKG2D and CD8 was observed after surgery in adjuvant-treated patients (Fig. 4c, d). Additionally, an increased percentage of NKp44^+^NKT-like cells was observed in non-adjuvant-treated patients after surgery (P* = 0.040, Fig. 4e). The expression level of NKp44 on NKT-like cells showed a similar pattern as the percentage of NKp44^+^NKT-like cells and was increased in non-adjuvant-treated patients after surgery (P* = 0.038, Fig. 4f). The percentage of NKp44^+^NKT-like cells, as well as the expression level of NKp44 on NKT-like cells, did not change in adjuvant-treated patients after surgery (Fig. 4e, f). In summary, as also observed for the immunophenotype of NK cells, NKT-like cells acquired expression of cell surface markers associated with functional activity after surgery in non-adjuvant-treated CC patients.

## Discussion

As shown in our recent study [12], the peripheral blood immune cell profile comprises a potential pool of biomarkers in CRC. We hypothesized that this profile might change upon therapeutic interventions. Therefore, we studied the peripheral blood immune profile in available postoperative PBMC samples (*N* = 24) from TNM stage II and III CC patients included in our previous study [12]. Our collection of PBMCs before and after tumor resection enabled, for the first time, to study the effects of tumor resection as well as adjuvant therapy on the subset distribution and immunophenotype of circulating immune cells in CC. Here, we focused on NK- and NKT-like cell subsets as several studies have reported an altered phenotype of these cells in peripheral blood of CRC patients compared to healthy donors, characterized by downregulation of activating receptors, which suggests impaired function of these immune cell subsets in cancer patients [12, 19, 20]. A critical role of the TME was implied in shaping NK- and NKT-like receptor-mediated anti-tumor immunity [15, 22, 23], which could be a result of hypoxic conditions, immunosuppressive cytokines or fibroblasts present in the TME [23–26].

We observed changes in peripheral blood immune cell profile after resection of the primary tumor in non-adjuvant-treated patients, including expansion of NKT-like (% of total PBMCs) and CD8^+^ T (% of total T cells) cell populations. An increase in percentage of these cell types was also observed after surgery in non-adjuvant-treated patients with laryngeal cancer [30]. Importantly, the reported expansion of the NKT-like and CD8^+^ T cell populations in the present study and that by Klatka et al. [30] is relative and does not provide information about a change in absolute numbers of these immune subsets. It was not possible to assess cells/ml counts in the present study since our samples were frozen PBMCs, not whole blood. Thus, trying to make measurements of cells/ml would be highly affected by the Ficoll procedure and sample preparation. This is a limitation of our study and should be taken into account when drawing conclusions. Grimm et al. [31] reported no absolute increase in CD8^+^ T cells in patients with oral squamous cell carcinoma (OSCC) after surgery, suggesting no effect on absolute immune cell numbers after surgery. However, it must be realized that this study was focused on OSCC patients that might have a different preoperative peripheral blood immune profile compared to CC patients which might also respond different to tumor resection. Furthermore, OSCC patients are often treated with neoadjuvant radiotherapy which might influence the immune system over a long period of time. The study by Grimm et al*.* did not specify neoadjuvant and/or adjuvant treatment of the included OSCC patients in their study. Up till now, studies reported on the numbers of circulating immune cells and subset distribution in cancer patients after surgery [30–32], but not on the immunophenotype of specific immune cell subsets. It is important that this is taken into account since the immunophenotype of immune cells is closely related to their function. Hence, expansion of the NKT-like cell population in CC patients after surgery does not necessarily mean that more effector cells are present. In this study, we showed that expression of activating receptors including NKG2D, NKp44 and CD8 was upregulated on NKT-like cells after surgery in non-adjuvant-treated patients. Additionally, we observed a decrease in the percentage of inhibitory receptor CD158a^+^ and NKG2A^+^ NKT-like cells, suggesting that the NKT-like cell population expands and acquires expression of cell surface markers associated with functional activity in CC patients after tumor resection. In contrast to studies on laryngeal cancer [30] and oral squamous cell carcinoma [31], we did not observe expansion of the NK cell population after tumor resection. We did, however, observe a change in immunophenotype of NK cells resembling the pattern observed in NKT-like cells. Hence, we observed upregulation of activating receptors NKG2D, NKp44 and CD8 on CD56^dim^ NK cells in non-adjuvant-treated CC patients after tumor resection. In line with this, upregulation of activating receptors NKG2D and CD8 was observed on CD56^bright^ NK cells as well as downregulation of the inhibitory receptor NKG2A after tumor resection in non-adjuvant-treated CC patients, suggesting that both CD56^dim^ NK and CD56^bright^ NK cells acquired a more active phenotype after tumor resection and, therefore, possibly recovered from TME-induced immunosuppression. The only activating marker that was downregulated in CC patients after tumor resection was the percentage of CD16^+^ cells within the CD56^bright^ NK cell population, suggesting differences in TME-induced recovery of CD56^dim^ and CD56^bright^ NK cells. This is in line with a previous study in which we also reported differences in immunophenotype of CD56^dim^ in comparison with CD56^bright^ NK cells in preoperative CRC patients [12], implicating different roles for NK cell subsets in patients, thereby emphasizing the need to analyze these subsets separately.

Several studies investigated short-term effects of surgical resection on the immune system in cancer patients (1–14 days after surgery) [32–36], whereas only a few studies focused on the long-term effects (≥ 6 weeks after surgery). Although these studies did not investigate the expression of different activating and inhibitory cell surface receptors, they all report expansion of cytotoxic immune cell subsets after surgery, including NK cells, NKT cells and CD8^+^ T cells [30–32], suggesting a more active immune system in patients after tumor resection, which is in line with our results. Importantly, the changes in immunophenotype of NK- and NKT-like cells observed in our study after surgery were restricted to patients that did not receive any adjuvant therapy, suggesting that adjuvant therapy delays or even prevents the recovery of TME-induced suppression of NK- and NKT-like cells. As the postoperative samples from adjuvant-treated patients were obtained ≥ 4 months after therapy completion, this implies long lasting immunosuppressive effects of adjuvant therapy. This is in line with a study in breast cancer patients that reported decreased numbers of CD4^+^ T cells and B cells even 9 months after completion of chemotherapy [37].

The fact that therapeutic interventions influenced the peripheral blood immune cell profile in CC patients has consequences regarding its biomarker potential. In an ideal situation, biomarkers such as circulating immune cells could be used to monitor treatment response and risk of recurrence in patients over time. Our data suggest that TME-induced immunosuppression is de-activated after resection of the primary tumor. In theory, patients without complete resection of the tumor or patients with micro-metastases after tumor resection still have a TME present which could mean that TME-induced immunosuppression of NK- and NKT-like cells is not removed. Despite the small sample size of our cohort and statistical correction for multiple testing, we still found significant results in our study. Further research is required to investigate whether the peripheral blood immune cell profile can be used as biomarker to determine the presence of residual tumor cells in cancer patients.

In conclusion, we observed expansion of NKT-like cells and CD8^+^ T cells and activation of both NK- and NKT-like cells after tumor resection in CC patients. This suggests TME-induced suppression of NK cells and NKT-like cells, an effect that could not be detected after surgical resection of the primary tumor. The changes in peripheral blood immune cell profile after tumor resection were restricted to patients that did not receive any adjuvant therapy, suggesting that adjuvant therapy delays or even prevents the recovery of TME-induced suppressed NK- and NKT-like cells in CC patients after surgery. These observations are of importance for using the peripheral blood immune cell profile as a biomarker in CC, but should also be considered when developing cancer immunotherapy for CC patients.

## Abbreviations

CC: Colon carcinoma
CRC: Colorectal cancer
DNAM-1: DNAX accessory molecule-1
HLA: Human leukocyte antigen
LUMC: Leiden University Medical Center
MFI: Median fluorescence intensity
NK: Natural killer
NKG2A: Natural killer group 2-A
NKG2C: Natural killer group 2-C
NKG2D: Natural killer group 2-D
NKT: Natural killer T
OSCC: Oral squamous cell carcinoma
PBMC: Peripheral blood mononuclear cell
TME: Tumor microenvironment
TNM: Tumor–node–metastasis
